# KETASER01 protocol: What went right and what went wrong

**DOI:** 10.1002/epi4.12627

**Published:** 2022-07-25

**Authors:** Anna Rosati, Manuela L’Erario, Roberto Bianchi, Sara Olivotto, Domenica Immacolata Battaglia, Francesca Darra, Paolo Biban, Annibale Biggeri, Dolores Catelan, Giacomo Danieli, Maria Cristina Mondardini, Duccio Maria Cordelli, Angela Amigoni, Elisabetta Cesaroni, Alessandra Conio, Paola Costa, Martina Lombardini, Rosanna Meleleo, Alessandra Pugi, Elena Eve Tornaboni, Marta Elena Santarone, Roberta Vittorini, Stefano Sartori, Carla Marini, Federico Vigevano, Massimo Mastrangelo, Silvia Maria Pulitanò, Francesca Izzo, Lucia Fusco

**Affiliations:** ^1^ Neuroscience Department Meyer Children’s Hospital‐University of Florence Florence Italy; ^2^ Intensive Care Unit Meyer Children’s Hospital‐University of Florence Florence Italy; ^3^ Intensive Care Unit Bambino Gesù Children’s Hospital, IRCCS Rome Italy; ^4^ Pediatric Neurology Unit Children’s Hospital Vittore Buzzi, ASST Fatebenefratelli Sacco Milan Italy; ^5^ Department of Child Neurology and Psychiatry Catholic University Rome Italy; ^6^ Child Neuropsychiatry Department of Surgical Sciences, Dentistry, Gynecology and Pediatrics University of Verona Verona Italy; ^7^ Department of Neonatal and Pediatric Intensive Care University Hospital Verona Italy; ^8^ Department of Cardiac, Thoracic, Vascular Sciences and Public Health University of Padua Padua Italy; ^9^ Department of Statistics, Computer Science, Applications G. Parenti University of Florence Florence Italy; ^10^ Department of Pediatric Anesthesia and Intensive Care Sant’Orsola‐Malpighi Hospital, University of Bologna Bologna Italy; ^11^ IRCCS Institute of Neurological Sciences of Bologna UOC Neuropsychiatry of the Pediatric Age Bologna Italy; ^12^ Intensive Care Unit, Department of Woman’s and Child’s Health University Hospital of Padua Padua Italy; ^13^ Child Neuropsychiatry Unit Polytechnic University of the Marche Ancona Italy; ^14^ Pediatric Intensive Care Unit Health and Science City Hospital‐University of Turin Turin Italy; ^15^ Department of Neuropsychiatry Ward Institute for Maternal and Child Health, IRCCS “Burlo Garofolo” Trieste Italy; ^16^ Intensive Care Unit Institute for Maternal and Child Health, IRCCS “Burlo Garofolo” Trieste Italy; ^17^ Clinical Trial Office Meyer Children’s Hospital‐University of Florence Florence Italy; ^18^ Association La Nostra Famiglia IRCCS Eugenio Medea Lecco Italy; ^19^ Department of Neuroscience Bambino Gesù Children’s Hospital, IRCCS, Full Member of European Reference Network EpiCARE Rome Italy; ^20^ Child and Adolescence Neuropsychiatry Unit Health and Science City Hospital‐University of Turin Turin Italy; ^21^ Pediatric Neurology Unit, Department of Woman’s and Child’s Health University Hospital of Padua Padua Italy; ^22^ Intensive Care Unit Catholic University Rome Italy; ^23^ Pediatric Intensive Care Unit Children’s Hospital Vittore Buzzi, ASST Fatebenefratelli Sacco Milan Italy

**Keywords:** children, non‐profit study, refractory status epilepticus, treatment

## Abstract

**Objective:**

To discuss the results of the KETASER01 trial and the reasons for its failure, particularly in view of future studies.

**Methods:**

KETASER01 is a multicenter, randomized, controlled, open‐label, sequentially designed, non‐profit Italian study that aimed to assess the efficacy of ketamine compared with conventional anesthetics in the treatment of refractory convulsive status epilepticus (RCSE) in children.

**Results:**

During the 5‐year recruitment phase, a total of 76 RCSEs treated with third‐line therapy were observed in five of the 10 participating Centers; only 10 individuals (five for each study arm; five females, mean age 6.5 ± 6.3 years) were enrolled in the KETASER01 study. Two of the five patients (40%) in the experimental arm were successfully treated with ketamine and two of the five (40%) children in the control arm, where successfully treated with thiopental. In the remaining six (60%) enrolled patients, RCSE was not controlled by the randomized anesthetic(s).

**Significance:**

The KETASER01 study was prematurely halted due to low eligibility of patients and no successful recruitment. No conclusions can be drawn regarding the objectives of the study. Here, we discuss the KETASER01 results and critically analyze the reasons for its failure in view of future trials.

**Key Points**

- Studies on the RCSE are difficult to be conducted, and not only for the rarity of the condition.
- Successful trial on RCSE requires emergency physicians, neurologists and intensivists are all sufficiently experienced in SE and familiar with the protocol.
- KETASER01 trial failure reasons are those commonly reported for non‐industry sponsorship studies.

## 1 INTRODUCTION

KETASER01 is a multicenter, randomized, controlled, open‐label, sequentially designed, non‐profit Italian study (ClinicalTrials.gov identifier: NCT02431663) that aimed to assess the efficacy of ketamine (KE) compared with conventional anesthetics in the treatment of refractory convulsive status epilepticus (RCSE) in children.^1^ The study was promoted and coordinated by Meyer Children’s Hospital‐University of Florence together with additional nine third‐level pediatric hospitals. By protocol, patients with RCSE unresponsive to first and second‐line drugs were randomized either to the experimental arm (KE up to 100 μg/kg/min) or to the control arm [midazolam (MDZ) up to 12 μg/kg/min and propofol (PR) up to 5 mg/kg/h and/or thiopental (TPS) up to 6 mg/kg/h]. The primary outcome was the EEG defined resolution of SE up to 24 hours after withdrawal of therapy. A secondary outcome was avoiding endotracheal intubation in the KE arm. Endotracheal intubation is a negative prognostic factor in SE,^2^, ^3^, ^4^ while cannot be avoided using TPS and PR, it may not be necessary when using KE.^5^ The assessment of this outcome made a double‐blind study design impossible. Adopting a sequential design with a non‐truncated triangular test, a sample size of 57 patients was estimated assuming 80% power, an α error of 5%, a success rate of 85% in the experimental arm and of 60% in the control arm.^6^ The estimation of the sample size was based on both the literature data on the efficacy of conventional and non‐conventional anaesthetics^7^, ^8^, ^9^, ^10^ and on our previous experience in treating RCSE with KE.^5^, ^11^ Although RCSE is a rare condition, the involvement of 10 participating Centers allowed us to consider the recruitment of the estimated sample size a feasible goal. According to the sequential trial design, we conducted the statistical analysis at each time a new patient had been recruited and the outcome measured. A sequential design consists of a series of interim analysis and stopping rules in order to stop the trial as soon as sufficient evidence in favor or against treatment would have been collected, maintaining a pre‐specified power and type I error probability level. A non‐truncated triangular test was adopted because we were uncertain about the magnitude of the treatment effect.

The KETASER01 trial was approved by the Italian Medicines Agency (October 2015) and by Ethics Committee. After 5‐year recruitment period, only 10 children were enrolled thus, following an interim analysis, we terminated the study on March 31, 2020 due to low eligibility of patients and no successful recruitment. Here we discuss the KETASER01 results and critically analyze the reasons for its failure in view of future trials.

## 2 METHODS

Patients were eligible for the KETASER01 study if (a) they were aged between 1 month to 18 years; (b) they presented with SE refractory to first‐line (oral or rectal benzodiazepines) and second‐line [phenytoin (PHT) 20 mg/kg or phenobarbital (PB) 20 mg/kg or both, plus MDZ up to 6 μg/kg/min] treatment; (c) their parents provided written consent. In order to guarantee the enrolment of a homogeneous population, the KETASER01 protocol also encompassed a well‐defined and standardized first‐ (when possible) and second‐line therapy before considering randomization to third‐line treatment. Patients with RCSE unresponsive to first‐line and second‐line drugs, if not already in paediatric intensive care unit (PICU), were transferred from the neurological department to the PICU and were randomized to the experimental or control arm by means of a computer‐assisted system. Block randomization was used with fixed size blocks and age stratification (<4.5 to 10 years and 11 to 18 years). Efficacy was defined as SE control up to 24 hours after the withdrawal of the anesthetic, associated with the following EEG features: (a) appearance of suppression‐burst pattern and/or; (b) appearance of widespread β activity and/or (c) appearance of slow activity in the absence of widespread or lateralized, continuous or sub‐continuous, and periodic abnormalities.

Anesthetics in both arms were infused continuously and titrated until RCSE resolution or a predetermined maximum dose of 100 μg/kg/min in the KE experimental arm. In the control arm, patients first received MDZ titrated until RCSE control or a predetermined maximum dose of 12 μg/kg/min. If RCSE continued, patients received PR, TPS, or both, titrated until SE control or a predetermined maxim dose (5 mg/kg/h and 6 mg/kg/h, respectively).^1^

Treatment failure was declared if RCSE persisted after the maximum treatment dose, if SE recurred while therapy was being tapered, or within 24 hours of its withdrawal, or due to withdrawal of the study drug owing to adverse events as defined according to the Common Toxicity Criteria for Adverse Events (CTCAE).^12^

The KETASER01 protocol was approved by the Italian Medicines Agency on January 7, 2015 and by the Tuscan Pediatric Ethics Committee (Coordinating Centre) on February 3, 2015. Approval dates from the local Ethics Committees of the nine participating hospitals ranged from May 21, 2015 to September 15, 2016. As stupefacient drugs, the supply of KE and MDZ required authorization from the National Ministry of Health. Each hospital had to ask for its own approval and the timing of the supply of the two stupefacient drugs, KE and MDZ, therefore varied among the Centers. The last step for each PICU at each Centre was to purchase the anesthetic drugs from local suppliers.

## 3 RESULTS

Only five of the 10 participating Centers, enrolled patients. The trial was halted on March 31, 2020 after enrolling only 10 children (five for each study arm; five females, mean age 6.5 ± 6.3 years) instead of the expected 57 patients. EEG defined resolution of SE up to 24 hours after withdrawal of therapy (primary outcome) was achieved in two of the five (40%) children enrolled in the experimental KE arm and in two of the five (40%) children in the control arm, where TPS was the effective anesthetic (odds ratio, 1.00; 95% CI (0.08, 12.56; *P* > .99)). In the remaining six patients, the randomized drug did not control RCSE. Clinical and demographic data for the 10 children are summarized in Table 1. In four of the 10 patients, SE was the presenting symptom in the context of an autoimmune (cases 2 and 6) and infective (cases 4 and 5) disease. In the remaining six patients, SE occurred in the context of their preceding epilepsy condition. During SE, seizures were focal motor in one case, focal to bilateral in five, generalized tonic–clonic in three and myoclonic in two. The duration of RCSE before randomization ranged from 1 hour to 7 days (median 12,50 hours; mean 42,75 hours) and it varied between the two groups (median 48 hours in the experimental arm vs median 11 hours in the control arm). First‐ and second‐line therapy failure was documented in all patients before randomization to the study (Table 2) and all underwent continuous EEG monitoring. Diffuse theta‐delta activity was observed in the two patients in whom KE was effective, while a suppression‐burst pattern was obtained in the two control arm children successfully treated with TPS (Table 3). Two patients, in whom the assigned treatment had failed, were switched to the other treatment arm, thereby exiting the protocol. One child (Table 2, case 10), after the inefficacy of MDZ and PR (control arm) was successfully treated with KE, while the second one (Table 2, case 6), following the inefficacy of KE in the experimental arm, was treated with MDZ, PR, and TPS that were all ineffective. The sequential analysis of three evaluations performed when enrolment included groups of six, eight, and 10 patients is shown in Figure S1. Table S1 shows the number of successes (RCSE resolution) in the experimental and control arms, the odds ratio, the score, the variance of the score, and the limits of the triangular test in the three evaluations for the grouping of six, eight, and 10 patients enrolled.

**TABLE 1:** Clinical and demographic data of 10 children enrolled in KETASER01 study

N°	Sex	Age	H/o epilepsy	Diagnosis of epilepsy	Type of SE	SE Etiology	Brain MRI during SE	Seizure types
1 Rome BGCH	M	12 y 11 m	Yes	IMIV	Remote Symptomatic	Rasmussen encephalopathy	Not performed	Focal
2 Rome BGCH	F	13 y 6 m	No	–	Acute Symptomatic	Anti GABA‐A Autoimmune Encephalitis	No cortical or subcortical alteration	Focal +**/−** SG
3 Florence MCH	M	8 m	Yes	CDG syndrome	Acute Symptomatic	Airway Infection	Not performed	GC
4 Milan Buzzi	F	3 m	No	–	Acute Symptomatic	Septic Shock	Not performed	GC
5 Milan Buzzi	F	4 m	No	–	Acute Symptomatic	Pneumococcal Meningitis	Not performed	GC
6 Florence MCH	M	9 y 8 m	No	–	Acute Symptomatic	FIRES	Bilateral HA	Focal +**/−** SG
7 Milan	M	9 y 4 m	Yes	17q21.31 microdeletion syndrome	Epilepsy related	No trigger	Not performed	Focal +**/−** SG
8 Rome BGCH	M	2 y 4 m	Yes	EE	Remote Symptomatic	GRIA3 encephalopathy	Not performed	MyS
9 Rome Gemelli	F	15 y 2 m	Yes	Post‐infective Focal Epilepsy	Acute Symptomatic	Infectious (Measles)	Multiple T2/FLAIR/DWI hyperintensity areas with slight enhancement in occipito‐parietal areas (right> left)	Focal +**/−** SG
10 Verona	F	8 y 2 m	Yes	Symptomatic Focal Epilepsy (cerebral palsy)	Remote Symptomatic	Airway infection	Not performed	MyS, Focal +**/−** SG

Abbreviations: BGCH, Bambino Gesù Children’s Hospital; CDG, Congenital Disorder of Glycosylation; EE, Epileptic Encephalopathy; F, female; FIRES, Febrile Infection‐Related Epilepsy Syndrome; GC, generalized convulsive; GRIA3, glutamate ionotropic receptor AMPA type subunit 3; HA, Hyppocampal Athrophy; H/o, History of; IMIV, Immune Mediated Inflammatory Vasculitis; M, male; m, months; MCH, Meyer Children’s Hospital; MyS, myoclonic seizures; SE, status epilepticus; SG, secondary generalization; y, years.

**TABLE 2:** Treatment regimen in ten children enrolled in KETASER01 study

N°	Baseline ASM treatment	First‐line Therapy	Second‐line Therapy	SE duration prior to randomization	KE^a^ (μg/kg/min) [D of I]	MDZ^a^ (μg/kg/min) [D of I]	PR^a^ (mg/kg/h) [D of I]	TPS^a^ (mg/kg/h) [D of I]	Efficacy (effective anesthetic)
1 Rome BGCH	CLB, PB, LCS, RUF	MDZ 0.08 mg/kg	MDZ 6 μg/kg/min	2 h 30 m	90 [5 d]	–	–	–	Yes (KE)
2 Rome BGCH	None	MDZ 0.2 mg/kg	PHT 20 mg/kg MDZ 6 μg/kg/min	7 d	100 [7 d]	–	–	–	Yes (KE)
3 Florence MCH	PB	DZP 0.1 mg/kg	MDZ 6 μg/kg/min	4 h	–	12 [48 d]	5 [1 d]	6 [5 d]	No
4 Milan Buzzi	None	MDZ 0.5 mg/kg	PHT 18 mg/kg PB 20 mg/kg MDZ 6 μg/kg/min	13 h	100 [4 h 30 m]	–	–	–	No
5 Milan Buzzi	None	DZP 0.5 mg/kg	PHT 20 mg/kg PB 10 mg/kg MDZ 6 μg/kg/min	1 h	–	12 [2 h]	–	3 [3 d]	Yes (TPS)
6 Florence MCH	None		PHT 20 mg/kg PB 20 mg/kg MDZ 6 μg/kg/min	2 d	100 [2 h]	–	–	–	No^b^
7 Milan Buzzi	CLB, VPA, TPM, LEV	DZP 0.4 mg/Kg MDZ 0.4 mg/kg	PB 20 mg/kg MDZ 6 μg/kg/min	11 h	–	–	–	4 [1 d]	Yes (TPS)
8 Rome BGCH	VPA, PB, LEV	MDZ 0.1 mg/kg	PHT 18 mg/kg PB 10 mg/kg MDZ 6 μg/kg/min	6 d	100 [3 h 30 m]	–	–	–	No
9 Rome Gemelli	CZP, LEV	MDZ 0.1 mg/kg	PHT 20 mg/kg MDZ 6 μg/kg/min	1 d	–	12 [15 m]	5 [40 m]	–	No
10 Verona	PB, LEV	MDZ 0.1 mg/kg	PHT 17 mg/kg MDZ 6 μg/kg/min	12 h	–	12 [15 d]	5 [5 h 30 m]	–	No^c^

Abbreviations: ASM, antiseizure medications; BGCH, Bambino Gesù Children’s Hospital; CLB, clobazam; CZP, clonazepam; d, days; DZP, diazepam; [D of I], Duration of Infusion; h, hours; KE, ketamine; LCS, lacosamide; LEV, levetiracetam; m, minutes; MCH, Meyer Children’s Hospital; MDZ, midazolam; PB, phenobarbital; PHT, phenytoin; PR, propofol; RUF, rufinamide; SE, status epilepticus; TPS, thiopental; VPA, valproate.

^a^ Maximum dose.

^b^ Midazolam (20 μg/kg/min), Propofol (7 mg/kg/h) and Thiopental (10 mg/kg/h), administered out of the protocol after the failure of the experimental arm treatment, were ineffective in controlling RCSE.

^c^ Ketamine, administered out of the protocol after the failure of the control arm treatment, was efficacious in controlling RCSE at the dosage of 100 μg/kg/min.

**TABLE 3:** EEGs patterns during RCSE and after third‐line treatment

N°	Distribution	Morphology	Third‐line treatment (Efficacy)	Pattern EEG at the RCSE control with the experimental and control drug
1 Rome BGCH	Focal	Focal fast activity	KE (Yes)	Diffuse theta‐delta activity
2 Rome BGCH	Focal bilateral	Bilateral asynchronous focal opercular fast activity	KE (Yes)	Diffuse theta‐delta activity
3 Florence MCH	Generalized	Spike and wave and polyspikes	MDZ/PR/TPS (No)	–
4 Milan Buzzi	Generalized	Rhythmic spike and waves and sharp waves	KE (No)	–
5 Milan Buzzi	Focal	Rhythmic spikes	TPS (Yes)	Burst‐suppression pattern
6 Florence MCH	Hemispheric shifting ictal activity	Prolonged focal fast activity	KE (No)	–
7 Milan Buzzi	Focal	Rhythmic continuous spike and waves	TPS (Yes)	Theta activity with focal abnormalities
8 Rome BGCH	Generalized	Spike and wave and polyspikes complexes	KE (No)	–
9 Rome Gemelli	Focal	Rhythmic Spike‐waves and polyspikes‐waves	MDZ/PR (No)	–
10 Verona	Generalized with left hemisphere predominance	Spike and waves, polyspikes	MDZ/PR (No)	–

Abbreviations: BGCH, Bambino Gesù Children’s Hospital; KE, ketamine; MCH, Meyer Children’s Hospital; MDZ, midazolam; PR, propofol; RCSE, Refractory convulsive status epilepticus; TPS, thiopental.

Endotracheal intubation was necessary in nine (90%) and avoided in one of the five patients receiving KE. The average length of stay in PICU was 26.5 days (range 7–65 days), 17.5 days (median) in the experimental group, and 10.5 days (median) in the control group. The average length of hospitalization was 70 days (range 15–285 days), 27 days (median) in the experimental arm, and 24 days (median) in the control arm (Table 4). Two patients, one from each group, required administration of intravenous inotropes, none of the 10 recruited children presented adverse events or death.

**TABLE 4:** Duration of ventilation and hospitalization

N°	Third‐line treatment	Efficacy	Duration of Ventilation (days)	Days of staying in PICU	Days of Hospitalization
1 Rome BGCH	KE	Yes	1	7	17
2 Rome BGCH	KE	Yes	23	28	37
3 Florence MCH	MDZ/PR/TPS	No	51	51	96
4 Milan Buzzi	KE	No	10	20	22
5 Milan Buzzi	MDZ/TPS	Yes (TPS)	8	13	22
6 Florence MCH	KE	No	60	65	98
7 Milan Buzzi	TPS	Yes (TPS)	6	8	26
8 Rome BGCH	KE	No	15	21	81
9 Rome Gemelli	MDZ/PR	No	29	39	285
10 Verona	MDZ/PR	No	7	13	15

Abbreviations: BGCH, Bambino Gesù Children’s Hospital; d, days; KE, ketamine; MCH, Meyer Children’s Hospital; MDZ, midazolam; PICU, pediatric intensive care unit; PR, propofol; TPS, thiopental.

During the recruitment phase, 400 SE was observed in the five active Centers, 191 of which were refractory based on the new SE classifications, that is, SE persisting despite administration of at least two appropriately selected and dosed parenteral medications including a benzodiazepine.^13^, ^14^ Of the 191 RCSE, 76 were treated with one or more third‐line anesthetics including the 10 enrolled in the study. In 66 RCSE, third‐line anesthetics were used out of the KETASER01 protocol. Reasons for the non‐enrolment of the 66 RCSE were as follows: (a) need of an urgent administration of anesthetic for endotracheal intubation (10 RCSE); (b) anesthetic already started in other hospitals before being transferred to third‐level Centre participating in the study (32 RCSE); (c) administration of second‐line drugs different from those reported in KETASER01 study and considered as a reason of exclusion from the study (12 RCSE); (d) medical decision not to enroll in the KETASER01 protocol due to previous history of SE refractory to anesthetics (3 RCSE) and sending to surgery for focal SE symptomatic of a cortical dysplasia (2 RCSE); (e) contraindications to the administration of one of the anesthetics (2 RCSE); (f) difficulties obtaining written informed consent from the both parents (1 RCSE); (g) patients previously treated with KE (2 RCSE); (h) patients already enrolled in KETASER01 study for a previous SE (2 RCSE).

## 4 DISCUSSION

The KETASER01 study was prematurely halted due to low eligibility of patients and no successful recruitment. After 5 years, only 10 patients of the 57 expected had been recruited. Only five Centers had been actively enrolling and not at the expected rate. Until the study was halted, no differences between KE (study arm) and MDZ, PR, and TPS (control arm) in terms of SE control and safety profile were observed. The results of this incomplete trial, including the evidence that, only four patients were successfully treated with the protocol therapy and randomization was only possible following failure to respond to MDZ infusion at the maxim dose of 6 μg/kg/min, prompt the concern that there might be a bias toward the selection of the most complex cases of an already severe condition.

No conclusions can be drawn regarding the objectives of the study nevertheless, we believe it is important to analyze and discuss the reasons behind the failure of the KETASER01 trial, particularly in view of future RCSE third‐line studies. We hypothesize that the failure of the study is ascribable to three main issues: (i) a too rigid protocol, (ii) the involvement of many different participating actors, that is, emergency department clinicians, neurologists, and intensivists, and (iii) its non‐profit nature.

Before KETASER01, SE treatment differed among the 10 participating Centers. The protocol envisaged a standardized, exclusive second‐line treatment with PB and/or PHT and MDZ up to 6 μg/kg/min before considering patient’s enrolment. To avoid false refractoriness of SE related to inappropriate treatment including type of drugs used and their doses, we chose to standardize the second‐line treatment. However, this decision resulted in the exclusion of 12 RCSE treated with second‐line drugs differing from that of the study protocol. Moreover, 42 individuals received anesthetics elsewhere before being transferred to the third‐level participating Centre (32 RCSE) or for urgent endotracheal intubation (10 RCSE). Thus, 54 patients were considered not eligible for KETASER01 study. A less rigid protocol might have allowed their enrolment, thus reaching 64 cases, beyond the target sample size of 57.

The management of RCSE requires the intervention of many different actors such as emergency specialists, pediatric neurologists, and intensivists.^15^, ^16^ This wide range of professionals involved would have required a broader and more accurate preliminary effort to establish an efficient recruiting network. The lack of involvement of local emergency networks and peripheral hospitals, places where the treatment is actually often started, may be considered another reason for KETASER01 failure.

Successful no‐profit trials are primarily dependent on the physician’s devotion to the idea of a potential benefit to the patient and his or her enthusiasm to commit time. Moreover, local structural, infrastructural, and procedural aspects in addition to lack of funding may affect investigators and represent barriers for conducting clinical trial. Industry‐sponsored trials have higher completion rates compared to trials sponsored by other sources.^17^ Non‐industry sponsorship, number of eligibility criteria, fewer study centers, and earlier trial phase have been recognized as the main reasons for the failure of clinical trials.^17^, ^18^, ^19^ KETASER01 is a non‐profit study, therefore, the advancement of each stage of the study was entrusted to the personal awareness and motivation of the individual professionals. Time for approval from the local Ethics Committees ranged from a minimum of one to a maximum of 20 months, two Centers never asked for experimental drug’s supplying by the National Ministry of Health and two others never bought the drugs. The lack of a contract research organization (CRO) was one of the main barriers to conduct our study.

Seven children displayed an extremely long duration of SE prior to randomization, ranging from 2 hours and 30 minutes to 7 days. The development of SE may be insidious and long‐lasting, with seizures that occur and become drug‐resistant over a period of hours and days, in spite of the use of anti‐seizure medications. This may be particularly true when SE occurs in an epileptic encephalopathy (EE), in which the underlying clinical conditions sometimes prevent assessment of the SE and a clear distinction between interictal and ictal discharges on EEG.^20^, ^21^, ^22^ Myoclonic SE can also at times be difficult to diagnose, especially when myoclonias are subtle and parcellar, as in our patient n = 8. Awareness of this condition may justify the decision to exclude EE from future SE clinical trials or to build a specific protocol study, considering a prompt and more aggressive treatment in these disorders.

Among clinical trials on SE, KETASER01 study first adopts a sequential design with a non‐truncated triangular test. Sequential analysis can be a useful and interesting tool in terms of time and resources, allowing for early stopping of a clinical trial.^6^, ^23^ This study design seems to be particularly helpful in the case of a comparison of a single experimental treatment with a single control arm, where the method works and provides satisfactory results. Status epilepticus, which is a rare condition with early outcome assessment, well fits to sequential design and we still suggest its application for new clinical trials. While there is a need to improve the current situation on the management of pediatric SE in Italy, it is also clear that a future study will need to be performed by at least 25–30 Centers over several years, with a dedicated, coordinated central management system and adequate funding.

Learning from the KETASER01 study, we built an Italian convulsive SE register as the *primum movens* for the development of diagnostic‐therapeutic pathways on a national scale and maybe a new clinical trial.

## CONFLICT OF INTEREST

None of the authors has any conflicts of interest to disclose.

## ETHICAL STATEMENT

We confirm that we have read the Journal’s position on issues involved in ethical publication and affirm that this report is consistent with those guidelines.

## Supporting information


Figure S1

Table S1
