# Supplementary material for: KETASER01 protocol: What went right and what went wrong
Source: Epilepsia Open. 2022 Jul 25;7(3):532–40. doi: 10.1002/epi4.12627 (PMC9436287; doi:10.1002/epi4.12627)
Supplement: Supplementary file 2 — Table S1 [file EPI4-7-532-s001.docx]

**Table 1S.** Number of successes for the treatment and control arm, the odds ratio, the score, the variance of the score and the two limits of the triangular test for the three evaluations at 6, 8, and 10 patients enrolled. The observed number of successes was 40% (95% CI 17-69%), against the hypothesized 60%.

| **Study Arm**  **(KE)** | **Control Arm**  **(MDZ, PR, TPS)** | **OR** | **sL** | **iL** | **upL** | **loL** |
| --- | --- | --- | --- | --- | --- | --- |
| 2/3 | 1/3 | 4 | 0.5 | 0.375 | 3.51 | -2.84 |
| 2/4 | 1/4 | 3 | 0.5 | 0.469 | 3.55 | -2.71 |
| 2/5 | 2/5 | 1 | 0.0 | 0.600 | 3.61 | -2.54 |
| Abbreviations: KE, ketamine; MDZ, midazolam; PR, propofol; TPS, thiopental. | | | | | | |
