# Supplementary material for: KETASER01 protocol: What went right and what went wrong
Source: Epilepsia Open. 2022 Jul 25;7(3):532–40. doi: 10.1002/epi4.12627 (PMC9436287; doi:10.1002/epi4.12627)
Supplement: Supplementary file 2 [file EPI4-7-532-s002.doc]

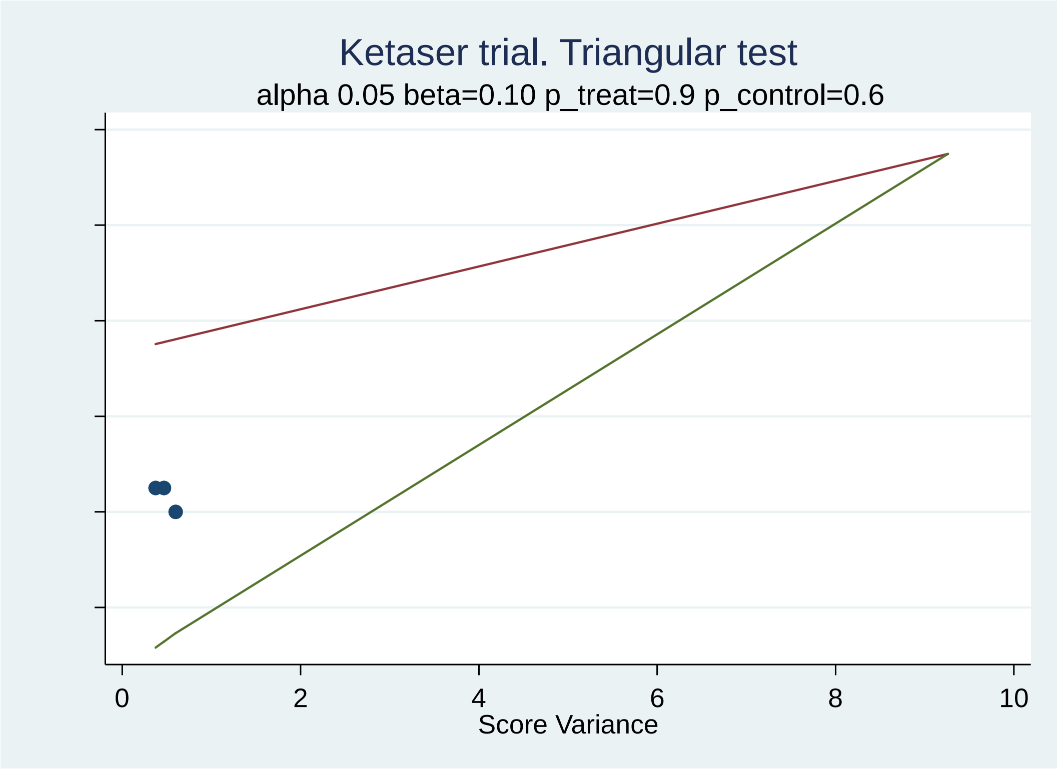
Ketaser trial. Triangular test
alpha 0.05 beta=0.10 p_treat=0.9 p_control=0.6
0
2
4
6
8
10
Score Variance
